# Immunobiotic Strains Modulate Toll-Like Receptor 3 Agonist Induced Innate Antiviral Immune Response in Human Intestinal Epithelial Cells by Modulating IFN Regulatory Factor 3 and NF-κB Signaling

**DOI:** 10.3389/fimmu.2019.01536

**Published:** 2019-07-03

**Authors:** Paulraj Kanmani, Hojun Kim

**Affiliations:** Department of Rehabilitation Medicine of Korean Medicine, Dongguk University Ilsan Hospital, Gyeongj-si, South Korea

**Keywords:** probiotics, PolyI:C, inflammatory response, antiviral immune response, immunoregulatory activity, intestinal epithelial cells

## Abstract

Many studies have demonstrated that immunobiotics with immunoregulatory functions improve the outcomes of several bacterial and viral infections by modulating the mucosal immune system. However, the precise mechanisms underlying the immunoregulatory and antiviral activities of immunobiotics have not yet been elucidated in detail. The present study was conducted to determine whether selected lactic acid bacteria (LAB) modulate toll-like receptor 3 (TLR3) agonist polyinosinic:polycytidylic acid (PolyI:C) induced viral response in human intestinal epithelial cells (IECs). PolyI:C increased the expression of interferon-β (IFN-β), interleukin-6 (IL-6), interleukin-8 (IL-8), monocyte chemoattractant protein (MCP-1), and interleukin-1β (IL-1β) in HCT116 cells, and these up-regulations were significantly altered when cells were pre-stimulated with LAB isolated from Korean fermented foods. LAB strains were capable to up-regulate IFN-β but down-regulated IL-6, IL-8, MCP-1, and IL-1β mRNA levels as compared with PolyI: C. HCT-116 cell treatment with LABs beneficially modulated the mRNA levels of C-X-C motif chemokine 10 (CXCL-10), 2-5A oligoadenylate synthetase 1 (OSA1), myxovirus resistance protein (MxA), TLR3, and retinoic acid inducible gene-I (RIG-I), and TLR negative regulators. In addition, LABs increased IFN-β, IFN-α, and interleukin-10 (IL-10) and decreased tumor necrosis factor-α (TNF-α) and IL-1β protein/mRNA levels in THP-1 cells. LABs also protected the cells by maintaining tight-junction proteins (zonula occludens-1 and occludin). The beneficial effects of these LABs were mediated via modulation of the interferon regulatory factor 3 (IRF3) and nuclear factor-kappa B (NF-κB) pathways. Overall, the results of this study indicate that immunobiotics have potent antiviral and anti-inflammatory activities that may use as antiviral substitutes for human and animal applications.

## Introduction

The gastrointestinal tracts (GITs) of humans and animals contain innate and adaptive immune cells that permit colonization by trillions of commensal microorganisms, which enhance digestion, and host mucosal immunity. Of the immune cells, intestinal epithelial cells (IECs) are the potent innate immune cells lined as a monolayer in the lumen of the GIT (1). These cells also act as a first line of defense against invading pathogens, including viruses such as rotaviruses (2, 3). IECs are able to sense and respond to various microbial stimuli from foreign and commensal microbiota via specialized surface membrane receptors such as toll-like receptors (TLRs) (1, 4). TLRs are a type of pattern recognition receptor (PRR), which have the ability to induce innate and adaptive immunity against invading pathogens by recognizing their molecular patterns (4). Among the TLRs, toll-like receptor 3 (TLR3) is able to recognize double stranded RNA (dsRNA) and to triggers intracellular signal transduction pathways in response to dsRNA viruses (5). After being ligated, TLR3 activates the transcription factors nuclear factor-kappa B (NF-κB) and interferon regulatory factor (IRF) via TLR adaptors molecules such as MyD88, Toll/interleukin-1 (IL-1) domain containing adaptor inducing IFN (TRIF), and TIRF-related adaptor molecule (TRAM), to produce inflammatory cytokines and interferons (IFNs) (6, 7). dsRNA is also recognized by cytosolic receptors such as retinoic acid inducible gene-I (RIG-1) and melanoma differentiation associated antigen 5 (MAD-5) (8, 9), which interact with IFN-β promoter stimulator-1 (IPS-1)/mitochondrial antiviral-signaling protein (MAVS) adaptor proteins and thus activate NF-κB and interferon regulatory factor 3 and 7 (IRF3, 7) to augment the expressions of inflammatory mediators and type I IFNs (7, 8). Collectively, previous reports suggest that IECs possess more than one receptor to sense dsRNA and its analog, and that they respond via two separate signaling pathways (7).

Polyinosinic:polycytidylic acid (PolyI:C) is a synthetic dsRNA analog that is often used to induce inflammatory responses that mimic response induced by dsRNA viruses (5). TLR3 and RIG-I/MDA-5 receptors have been reported to be able to recognize PolyI:C and to activate transcription factors responsible for the expressions of inflammatory cytokines/chemokines and type I IFNs (7, 10). The production of IFNs, especially of type I IFNs, plays a crucial role in protecting host immune system from viral invasion. In particular, IFN-β has the ability to inhibit viral replication (7). The absence of IFN-β in mice was highly infected by viruses (11). In addition, the activation of type I IFN signaling induces the expression of several antiviral genes, such as myxovirus resistance protein (MxA), and 2′-5′ oligoadenuylate dependent endoribonuclease (RNase-L), that helps maintain antiviral states induced by IFNs in hosts via several mechanisms (12, 13).

Lactic acid bacteria (LAB), a group of commensal bacteria that are able to exert probiotic effects by mutually interacting with host IECs (14). Lactobacilli and bifidobacteria are the members of LAB, which are dominantly colonizing in the GIT and boost the host immune system to combat viral infections (15). Several studies evaluated the beneficial actions of these strains against viral and PolyI:C-mediated inflammatory responses (3, 16–18). In addition, the extracellular polysaccharides (EPS) of these probiotic strains have also been reported to promote host defense mechanism and to attenuate inflammatory responses induced by pathogens or PolyI:C (3, 19, 20). Most studies used IECs (Human and Porcine IECs) as an *in vitro* model to study innate anti-viral immune response of LAB strains against rotavirus (RV) and PolyI:C (21–23). Human intestinal epithelial (HT-29) cells potently respond to PolyI:C by up-regulating immune gene proteins related to the TLR signaling pathway (10), and a study using porcine jejunal cells (IPEC-J2) showed treatment with *L. rhamnosus* GG reduced inflammatory response and RV infection *in vitro* (22). Also, PolyI:C increased the mRNA level of inflammatory cytokines (IL-6, IL-8, MCP-1) and interferon (IFN-α and IFN-β) in porcine IECs (PIE cells) (17, 23), whereas PIE cells treated with immunobiotic *L. casei* MEP221106 up-regulated IFN-α and IFN-β and down-regulated IL-6 and MCP-1 in response to the TLR3 agonist PolyI:C (16). These studies also suggest that IECs are the *in vitro* useful model to select and study of probiotic bacteria against viral or PolyI:C induced immune response *in vitro*. In addition, IECs would helpful to study molecular insight into mechanisms involved in the viral and anti-viral response of PolyI:C and probiotic strains via analysis of TLRs expression, activation, and modulation of innate immune signaling pathways and negative regulatory proteins. In the present study, we used human colon cell line (HCT116) to investigate the antiviral effects of probiotic bacteria isolated from Korean foods. To induce viral response, HCT116 cells were treated with PolyI:C and then examined for changes in the expressions of inflammatory cytokines, IFNs, anti-viral proteins, and TLR negative regulators. In addition, modulations of tight-junction proteins (ZO-1 and occludin) and signaling molecules (IRF-3 and IκB-α,) were also examined by western blotting after treating cells with *Lactobacillus plantarum, Weissella cibaria*, or *Lactobacillus sakei*.

## Materials and Methods

### Bacterial Strains

The strains *L. plantarum* DU1, *W. cibaria* DU1, and *L. sakei* DU2 used in this study were previously isolated from Korean fermented foods and maintained in MRS (deMan-Rogosa-Sharp) medium at −70°C. LABs were grown at 37°C for 19 h, centrifuged, washed with distilled phosphate buffered saline (PBS), and re-suspended in Roswell Park Memorial Institute 1640 medium (RPMI 1640, Gyeongsagbuk-do, South Korea) at desired concentrations, then stored at −4°C until required. The cytotoxicity of these strains on human cell line was determined previously using a cell viability assay kit (EZ-CYTOX, DOGEN Bio Co. Ltd) (24).

### Cell Culture

The human colon and monocytic cells (HCT116 cells and THP-1) were used in this study that were obtained from the Korean Cell Line Bank (Seoul). HCT116 cells were cultured in RPMI medium supplemented with 10% fetal bovine serum (FBS), and 1% penicillin/streptomycin (P/S) at 37°C, under 5% CO_2_. The medium was changed at 1-day interval for 5–6 days. Cells from passages 20–40 were used in the present study. In addition, THP-1 cells were also cultured in RPMI-1640 medium containing FBS (1%), P/S (1%), and mercaptoethanol (0.05 mM) at 37°C, under 5% CO_2_ for 5–6 days. To induce differentiation, cells were incubated with PMA (phorbol-12-myristate-13-acetate) medium for 48 h and then in fresh RPMI medium for 24 h. PMA medium was prepared by adding PMA (50 ng/ml) to RPMI medium.

### Analysis of Antiviral Activity of LABs in HCT116 Cells

HCT116 cells (3 × 10^4^ cells/ml) were placed in collagen coated plates (SPL Life Sciences Co. Ltd, Gyeonggi-do, Korea), and incubated at 37°C, under 5% CO_2_ for 5–6 days. Cultured cells were then incubated with LAB strains (5 × 10^7^ cells/ml) for 48 h and post-incubated with PolyI:C (10 μg/ml) for 3 or 12 h. HCT116 cells stimulated with PolyI:C and medium alone were used as positive and negative controls, respectively. The RNA was extracted from treated cells and the expressions of type I interferon (IFN-α, IFN-β), antiviral proteins [(MxA, OAS1, C-X-C motif chemokine 10 (CXCL-10)], signaling receptors (RIG-I, TLR3), inflammatory cytokines/chemokine (IL-6, IL-8, MCP1, IL-1β), and TLR negative regulators such as A20, toll-interacting protein (Tollip), single Ig interleukin 1 –related receptor (SIGIRR) and IL-1 receptor-associated kinase-M (IRAK-M) were analyzed by qRT-PCR.

### RNA Extraction and Quantitative Polymerase Chain Reaction (qPCR)

Total RNA was isolated from cells by adding TRIzol reagent (Invitrogen), and used to synthesize cDNA using a Thermal cycler (BioRad, Hercules, CA, USA). qPCR was performed with a 7300 real-time PCR system (Roche Applied Science, Indianapolis, IN, USA) using SYBR green and targeted primers (24). PCR reaction mixtures (20 μl) contained 1 μl of cDNA and 19 μl of master mix, which included SYBR green and forward and reverse primers (1 pmol/μl). Amplifications were performed using the following procedure; 95°C for 5 min, followed by 40 cycles of 95°C for 15 s, 60°-63°C for 30 s, and 72°C for 30 s. β-actin was used as the internal control to normalize cDNA levels.

### Co-culture Study

HCT116 cells (3.5 × 10^4^ cells/well) were cultured in apical transwell culture inserts (transparent PTFE membrane coated collagen (0.4 μm pore size); Transwell-COL, Corning Inc., NY, USA) at 37°C under 5% CO_2_ for 5–6 days. Then, HCT116 cells were co-cultured with THP-1 cells (1 × 10^5^ cells/well) taken in a basolateral culture chamber. To examine the anti-viral immune response of LABs, HCT116 cells monolayer [Transepithelial electric resistance (TEER value ~541 Ω cm2)] was stimulated with LABs for 48 h, after which 10 μg/ml of PolyI:C was added to the THP-1 cells cultured chamber and incubated for an additional 12 h at 37°C. Cell free supernatants from the basolateral chamber was collected and stored at −4°C to estimate the protein level of tumor necrosis factor-α (TNF-α). In addition, RNA from THP-1 was used to analyze the expression of IFN-α, IFN-β, IL-10, and IL-1β by qRT-PCR.

### Enzyme-Linked Immunosorbent Assay (ELISA)

To determine whether LABs reduced TNF-α production in the co-culture model, TNF-α levels in THP-1 cell free supernatants from basolateral sides were quantified using a commercially available ELISA kit (Human TNF-α Quantikine ELISA kit, R & D system, MN, USA).

### Proteins Extraction and Western Blot Analysis

HCT116 cells (1.8 × 10^5^ cells/dish) were seeded in dishes (60 mm) and incubated at 37°C under 5% CO_2_ for 5–6 days. Fully confluent cells were then stimulated as follows; cells stimulated with LAB strains alone (48 h), cells stimulated with PolyI:C alone (2 h), cells pre-stimulated with LAB strains and combined with PolyI:C for last 2 h (PolyI:C combined 2 h), cells pre-stimulated with LAB strains and post-stimulated with PolyI:C for 2 h (2 h, PolyI:C post-treatment separately), and cells co-stimulated with LAB strains +PolyI:C (48 h both combined). Treated cells were washed three times with distilled PBS and lysed with 200 μl of CellLytic M cell lysis reagent (Sigma-Aldrich, St. Louis, MO) containing phosphatase and protease inhibitors. Lysed cells were scraped and transferred to fresh Eppendorf tubes (1.5 ml), sonicated at 50% for 3–5 s, and stored at −70°C until required. Total protein in collected samples was estimated using bicinchoninic acid (BCA) assay kits (Thermo Scientific, Pierce, Rockford, IL, USA) after heating samples at 95°C for 5 min.

For western blotting, lysed samples were loaded in 10% SDS-polyacrylamide gels, and separated proteins were transferred to nitrocellulose membranes (Trans-Blot Turbo™, BioRad) that were incubated with blocking buffer for 1–2 h and then incubated with targeted proteins specific primary and secondary antibodies. Tight junction proteins (zonula occludens-1, occludin), phosphorylation of interferon regulatory factor 3 (p-IRF3) and nuclear factor kappa B (p-IκB-α) levels were evaluated by incubating membranes overnight with ZO-1 (D7D12) antibody (ZO-1, Cat. #8193), phosphor-IRF3 (Ser396) antibody (p-IRF3, Cat. #29047), phospho-IκB-α antibody (p-IκB-α, Cat. #2859) (Cell Signaling Technology, Beverly, MA, USA), occludin antibody (E-5: Cat.#SC-133256), and β-actin antibody (C4, Cat. #SC-47778) (Santa Cruz Biotechnology Inc., Dallas, Texas) at dilutions of 1,000:1. Membranes were then washed with TBS-T buffer and incubated with Goat anti-rabbit IgG-HRP polyclonal antibody (AbFrontier, Cat. #LFSA8002, Seoul). After 1–2 h of incubation, membranes were washed with TBS-T buffer, and treated with western blot detection solution (Dyne ECL Star, Korea). The optical protein bands were detected and the densitogram peaks were estimated using the Image J software (National Institute of Health, Bethesda, MD, USA).

### Statistical Analysis

The data were expressed as the average (mean ± SD) value of three repeated experiments. Significant differences among the groups were determined by one-way analysis of variance (ANOVA) with Tukey multiple range test using SPSS ver. 12.0 (SPSS Inc., Chicago, IL, USA). Statistical significance was accepted for *p* values of < 0.05.

## Results

### LABs Modified PolyI:C Induced IFN-β Expression in HCT116 Cells

**Figure 1:**
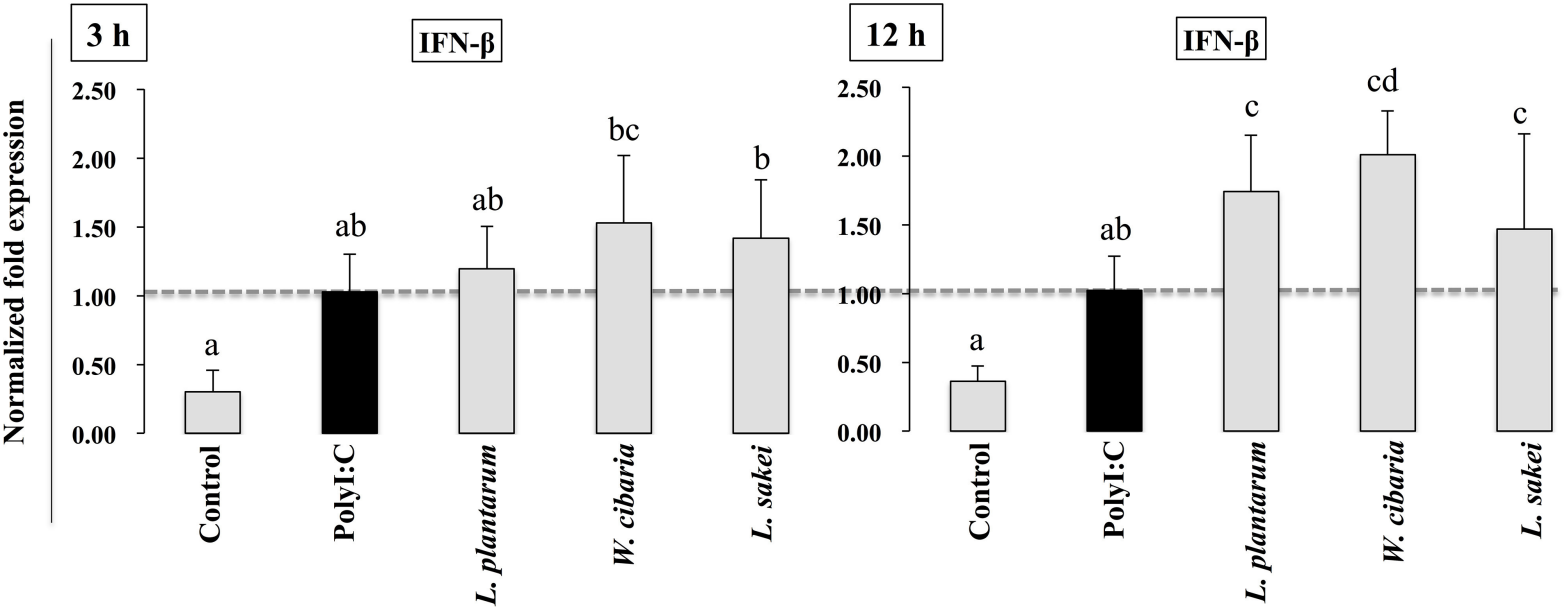
LAB strains up-regulate PolyI:C induced IFN-β in IECs. After HCT116 were treated with LAB strains and PolyI:C, the expression of IFN-β was determined by RT-PCR. Cells treated with either PolyI:C or medium alone were used as positive and negative controls, respectively. The positive control was used for comparison of LAB strains treated groups. The mean differences among the different superscript letters (a, b, ab, cd) were significant at 0.05 level. LABs were able to up-regulate IFN-β level in response to PolyI:C.

We first evaluated whether the three LAB strains (*L. plantarum* DU1, *Weissella cibaria* DU1, and *L. sakei*) induced IFN-β production in response to PolyI:C in HCT116 cells. Cells were pre-incubated with LABs and then post-stimulated with PolyI:C for different hours. The results of RT-PCR showed that the expression of IFN-β varied depending on the stimulation hours. Stimulation of cells with PolyI:C alone increased the expression of IFN-β at both 3 and 12 h (Figure 1). The PolyI:C induced IFN-β expression was further up-regulated when cells were pre-incubated with LABs. *W. cibaria* and *L. sakei* were significantly (*p* < 0.05) increased the level of IFN-β in 3 h, whereas *L. plantarum* showed level was not significantly higher than PolyI:C. But at 12 h, all LAB strains significantly (*p* < 0.05) increased the mRNA level of IFN-β in response to PolyI:C in HCT116 cells, indicating that LABs showed strong antiviral activity against PolyI:C at late stage of stimulation.

### Effect of LABs on Inflammatory Cytokine/Chemokine Expressions in HCT116 Cells

**Figure 2:**
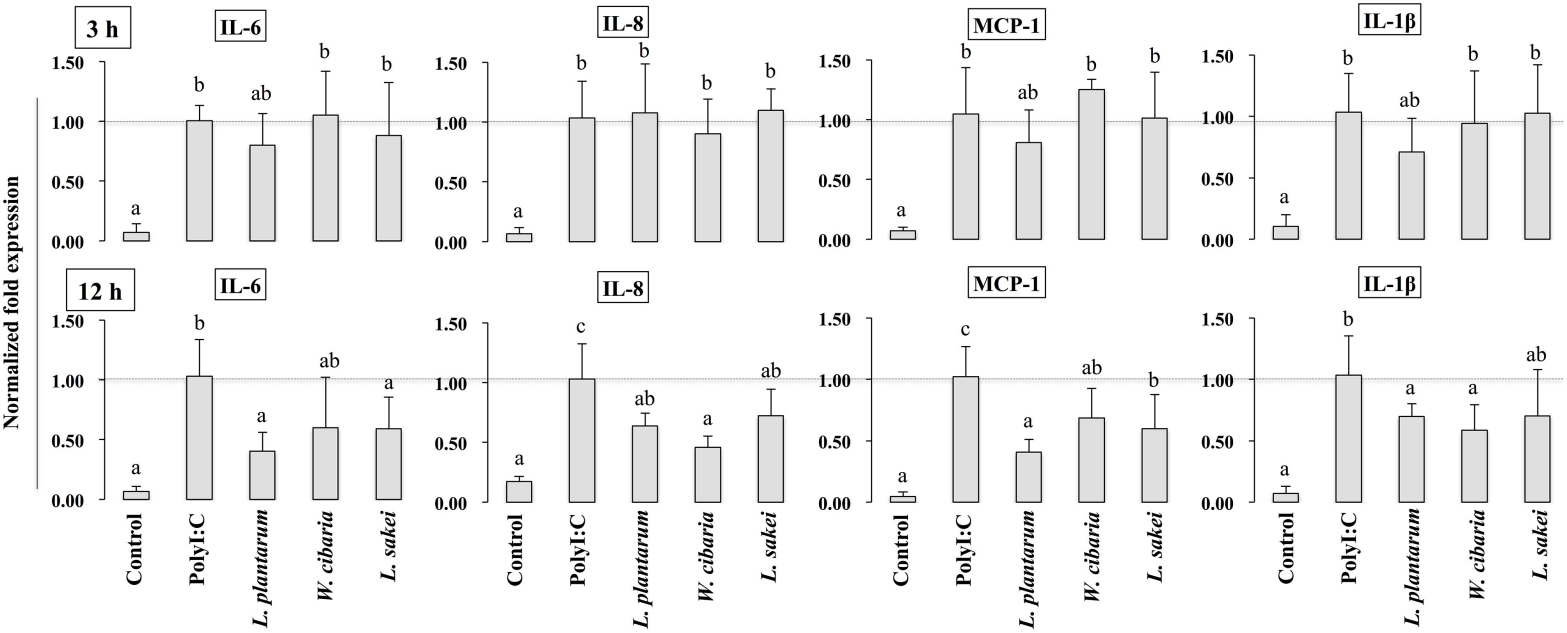
LAB strains attenuate the inflammatory response triggered by TLR3 in IECs. After HCT116 cells were treated with LAB strains and PolyI:C, the expression of IL-6, IL-8, MCP-1, and IL-1β were analyzed by RT-PCR. Cells treated with either PolyI:C or medium alone were used as positive and negative controls, respectively. The positive control was used for comparison of LAB strains treated groups. The mean differences among the different superscript letters (a, b, ab) were significant at 0.05 level. LABs decreased the expression of PolyI:C induced inflammatory cytokines in HCT116 cells.

To investigate whether LABs inhibit PolyI:C-induced inflammatory cytokine/chemokine expressions, HCT116 cells were pretreated with LABs and then with PolyI:C, as described above. As shown in Figure 2, PolyI:C treatment tended to increase the mRNA levels of IL-6, IL-8, MCP-1 and IL-1β, but LABs pretreatment altered these expressions in a time-dependent manner. At 3 h, cells pre-stimulated with LABs altered the expression of all cytokines, however they were relatively similar to the levels of PolyI:C (Figure 2). In contrary, all LAB strains had profound effects on the reduction of IL-6, IL-8, MCP-1, and IL-1β at 12 h, and these reductions were significantly (*p* < 0.05) lower than PolyI:C.

### LABs Altered TJ Proteins in HCT116 Cells

**Figure 3:**
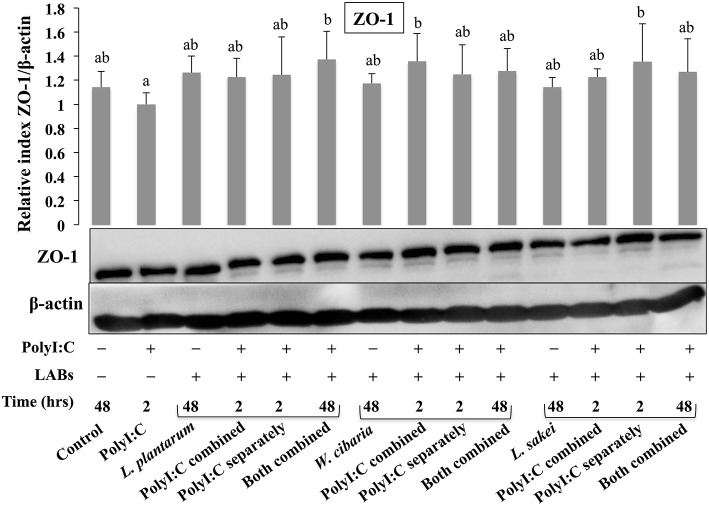
LAB strains modulate TJ protein (ZO-1) in HCT116 cells. Cells were stimulated as follows: control, PolyI:C alone, LABs alone (48 h), LABs+PolyI:C (2 h, Poly:C combined treatment), LABs+PolyI:C (2 h, PolyI:C post-treatment separately), and or LABs+PolyI:C (48 h both combined). The level of tight-junction protein (ZO-1) was analyzed by western blot. The loading control beta-actin was reused for illustrative purposes. The bar graphs represent the results of three independent experiments. The mean differences among the different superscript letters (a, b, ab) were significant at 0.05 level. LABs stimulation modulated ZO-1 level in response PolyI:C.

**Figure 4:**
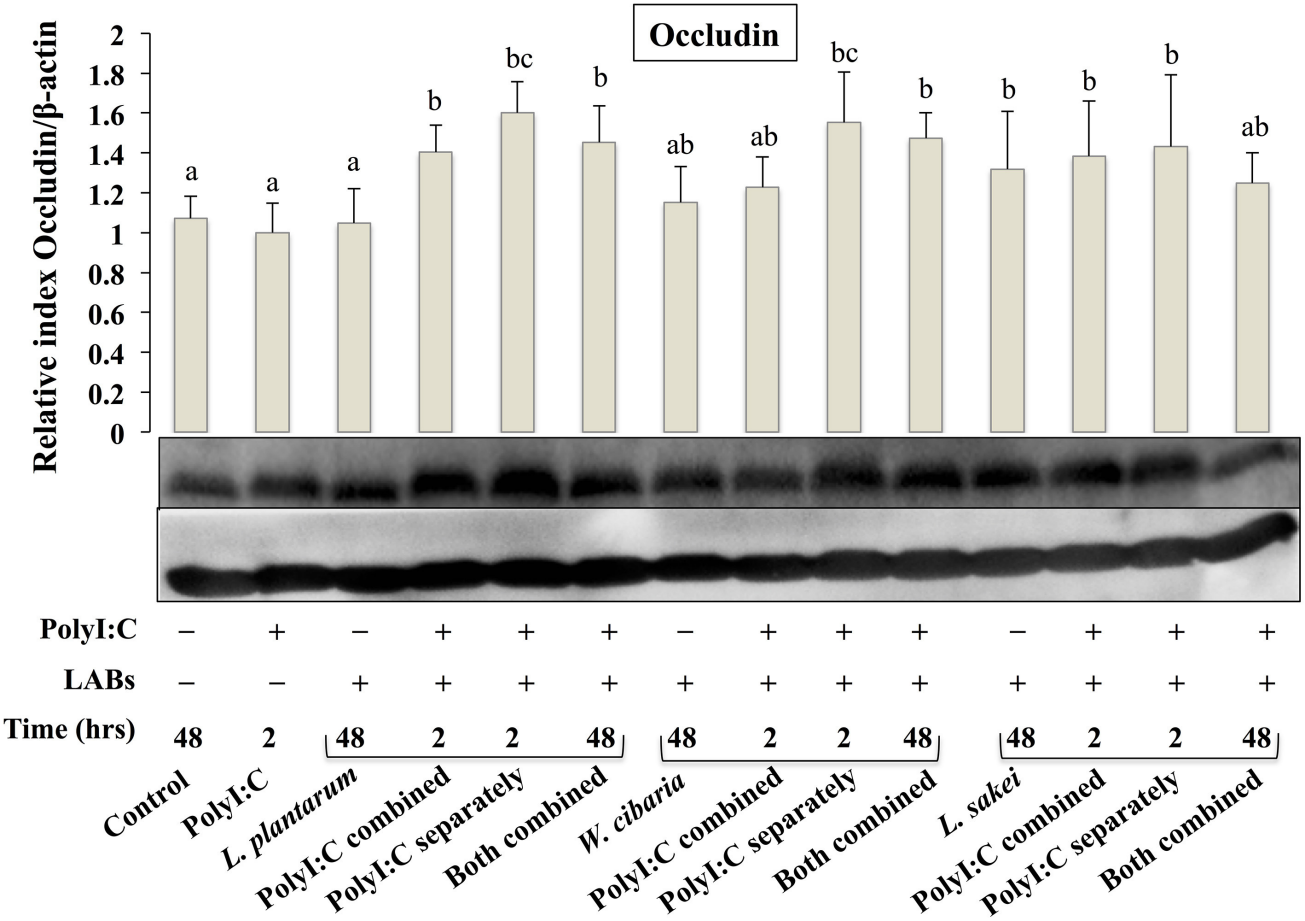
LAB strains modulate TJ protein (Occludin) in HCT116 cells. Cells were stimulated as follows: control, PolyI:C alone, LABs alone (48 h), LABs+PolyI:C (2 h, Poly:C combined treatment), LABs+PolyI:C (2 h, PolyI:C post-treatment separately), and or LABs+PolyI:C (48 h both combined). The level of TJ protein (ZO-1) was analyzed by western blot. The loading control beta-actin was reused for illustrative purposes. The bar graphs represent the results of three independent experiments. The mean differences among the different superscript letters (a, b, ab, c, bc) were significant at 0.05 level. LABs alone or co-treated with PolyI:C increased the level of occludin in HCT116 cells.

To analyze the effect of LABs on alternation of TJ, we examined the level of ZO-1 and occludin proteins in HCT116 cells that were pre or co-stimulated with LAB strains and PolyI:C or PolyI:C alone. HCT116 cells treated with PolyI:C decreased the level of ZO-1, and this decrease was attenuated by pre or co-treated with LAB strains (Figure 3). As compared to PolyI:C, all LABs except *L. plantarum* and *W. cibaria* showed significantly higher level of ZO-1 in HCT116 cells post-treated with PolyI:C for 2 h. In addition, higher level of ZO-1 was observed in HCT116 cells co-stimulated with LABs and PolyI:C for 48 h. Relatively, similar pattern of results were observed in the protein level of occludin (Figure 4). LABs treatment increased occludin protein in HCT116 cells that were post-stimulated with PolyI:C for 2 h. In addition, the occludin was found to be higher in cells that were co-stimulated with LABs and PolyI:C for 2 and 48 h. These results indicates that LABs protect the cells against PolyI:C by maintaining the tight-junction proteins.

### LABs Modulated PolyI:C-Induced Cytokine and Antiviral Protein Expressions in HCT116 Cells

**Figure 5:**
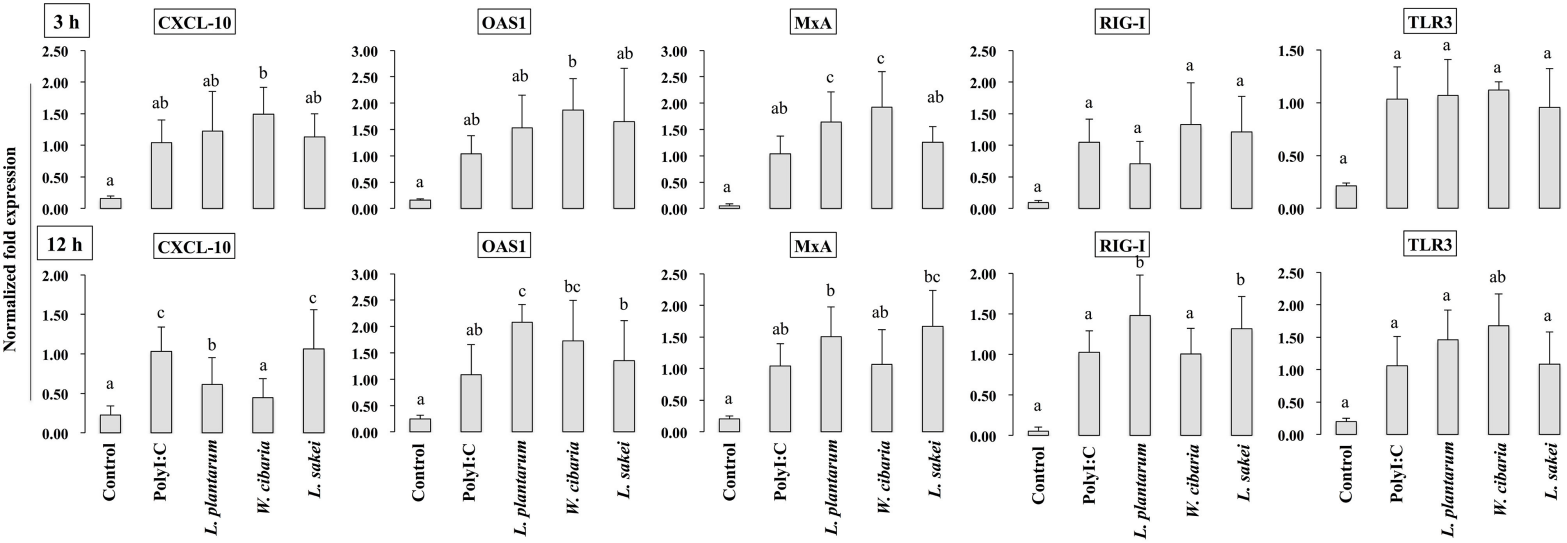
LABs modulate expressions of antiviral proteins and receptors in HCT116 cells. After HCT116 cells stimulated with LABs and PolyI:C for 3 and 12 h, the mRNA level of CXCL-10, OAS1, MxA, RIG-I, and TLR3 was evaluated by RT-PCR. Cells treated with either PolyI:C or medium alone were used as positive and negative controls, respectively. The positive control was used for comparison of LAB strains treated groups. The mean differences among the different superscript letters (a, b, ab, c, bc) were significant at 0.05 level. LABs stimulation decreased the level of CXCL-10, while increased level of other anti-viral proteins at 12 h in HCT116 cells, post-treated with PolyI:C.

HCT116 cells were treated with LABs and followed by post-stimulation with PolyI:C for 3 or 12 h. Relative mRNA levels of cytokines, antiviral proteins, and signaling receptors were determined by RT-PCR. CXCL-10 levels were significantly increased in HCT116 cells treated with PolyI:C for 3 or 12 h (Figure 5). Pre-stimulation of cells with LABs modulated PolyI:C induced cytokine mRNA levels. *L. plantarum* or *W. cibaria* pretreatment increased CXCL-10 level after 3 h of PolyI:C post-treatment, whereas *L. sakei* pretreatment had no effect. However, pretreatment with all three LABs up-regulated mRNA levels of the antiviral proteins (OAS1 and MxA) as compared with PolyI:C. The expression of RIG-I and TLR3 weren't significantly increased by LAB strains as compared with PolyI:C. In contrary, the expression of CXCL-10 was significantly (*p* < 0.05) decreased when cells were pre-stimulated with LABs except *L. sakei* for 12 h, whereas the expressions of OAS1 and MxA were increased by LABs (except *W. cibaria* for MxA) in HCT116 cells (Figure 5). In addition, *L. plantarum* and *L. sakei* were significantly increased the level of RIG-I in HCT116 cells. The mRNA level of TLR3 was significantly increased when cells were pre-treated with *W. cibarai* as compared with PolyI:C.

### Effect of LABs on TNF-α Production in THP-1 Cells

**Figure 6:**
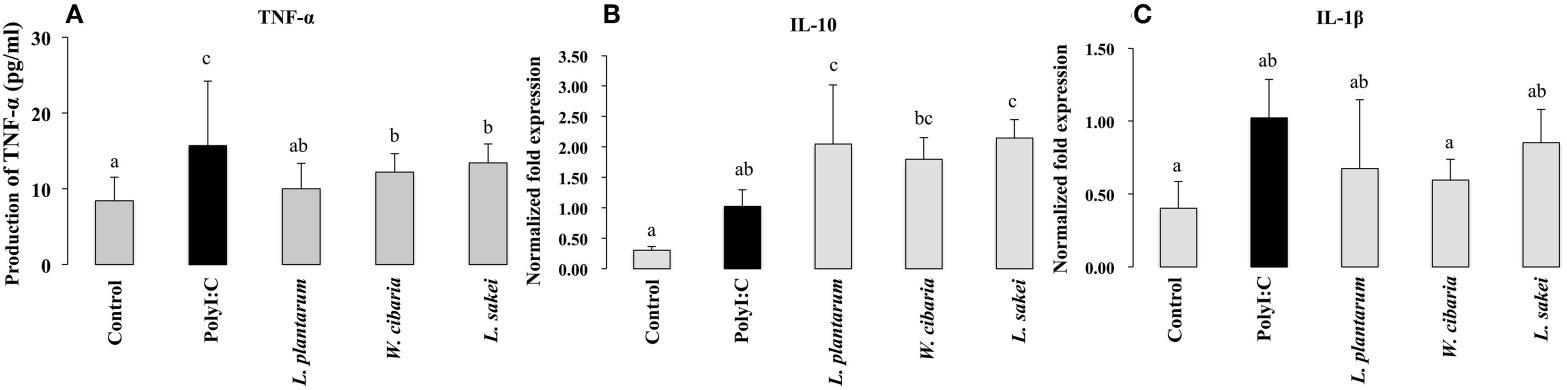
Effect of LABs on modulation of inflammatory and anti-inflammatory cytokines expression in THP-1 cells cultured with HCT116 cells. After HCT116 cells were stimulated with LABs, and followed by THP-1 cells with PolyI:C for 12 h, the production of TNF-α in the basolateral medium and mRNA level of IL-10 and IL-1β in THP-1 cells was analyzed by ELISA and RT-PCR. Cells treated with either PolyI:C or medium alone were used as positive and negative controls respectively The positive control was used for comparison of LAB strains treated groups. The mean differences among the different superscript letters (a, b, ab, c, bc) were significant at 0.05 level. LABs stimulation decreased the level of TNF-α, IL-1β, while increased level of IL-10 in THP-1 cells, post-treated with PolyI:C.

To investigate the effect of LABs on TNF-α production, we used a co-culture model mimicking intestinal conditions by allowing cell crosstalk via the secretions of soluble factors into the surrounding medium. HCT116 cells were co-cultured with THP-1 cells and stimulated with LABs and followed by PolyI:C for 12 h. TNF-α levels in medium were determined by ELISA. Results are shown in Figure 6A. PolyI:C treatment tended to increase the production of TNF-α in THP-1 cells; however, LABs pre-stimulation suppressed THP-1 cells to produce lower level of TNF-α as compared to PolyI:C treatment alone.

### LABs Modulated PolyI:C-Induced IFNs and Inflammatory and Anti-inflammatory Cytokines in THP-1 Cells

**Figure 7:**
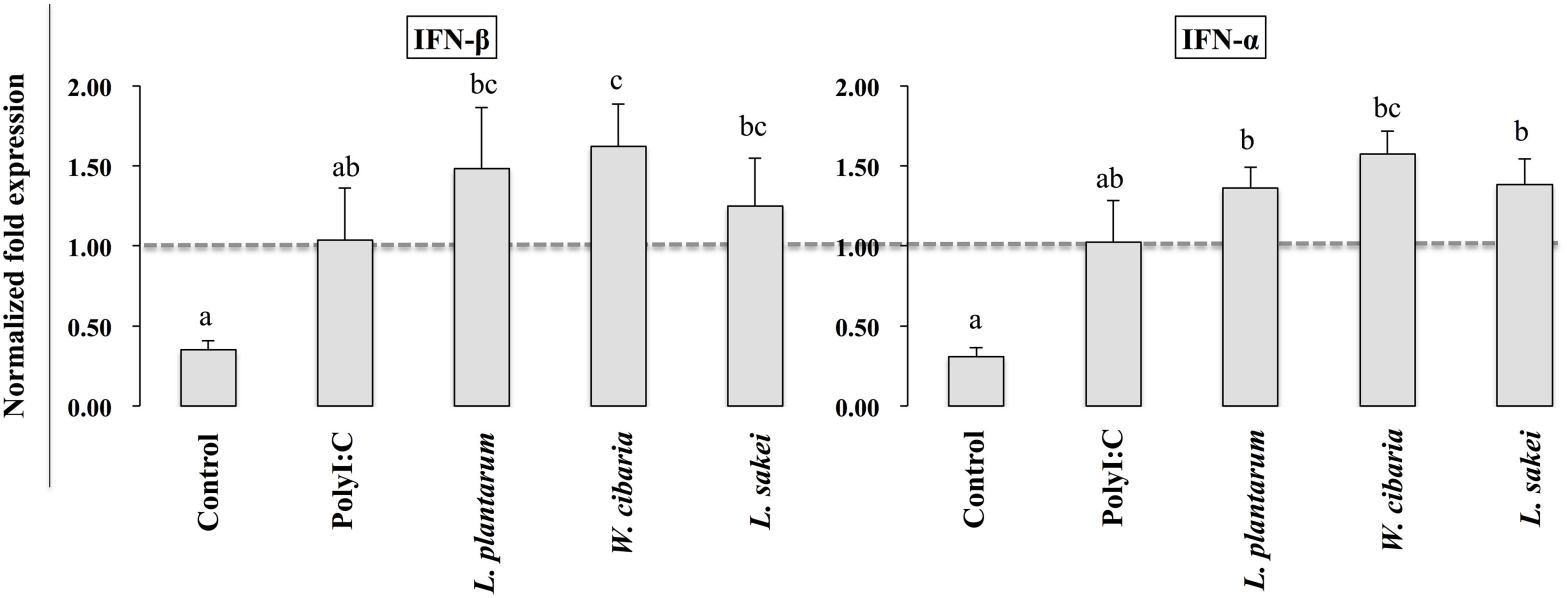
Effect of LABs on up-regulation of antiviral IFNs in THP-1 cells cultured with HCT116 cells. The expression of IFN-α and IFN-β were analyzed by RT-PCR. Cells treated with either PolyI:C or medium alone were used as positive and negative controls, respectively. The positive control was used for comparison of LAB strains treated groups The mean differences among the different superscript letters (a, b, ab, c, bc) were significant at 0.05 level. LABs stimulation increased the level of IL-1β and IFN-α in response to PolyI:C THP-1 cells.

To investigate whether LABs indirectly modulate PolyI:C induced type 1 IFNs and cytokines in THP-1 cells via HCT116 cells, we extracted RNA from THP-1 cells that were stimulated with LABs and PolyI:C. The expression of interleukin-10 (IL-10), interleukin 1β (IL-1β), IFN-α, and IFN-β was analyzed by qRT-PCR. The mRNA level of IL-10 was significantly increased by all three LABs in THP-1 cells post-stimulated with PolyI:C for 12 h. On the other hand, *W. cibaria* exhibited significant reduction in the level of IL-1β in THP-1 cells (Figures 6B,C). *L. plantarum* and *L. sakei* did not significantly diminish IL-1β expression as compared to PolyI:C. Stimulation of THP-1 cells with PolyI:C increased the expressions of IFN-α and IFN-β and these expressions were further increased by LABs post-stimulation (Figure 7).

### LABs Modulated the Expressions of TLR Negative Regulators in HCT116 Cells

**Figure 8:**
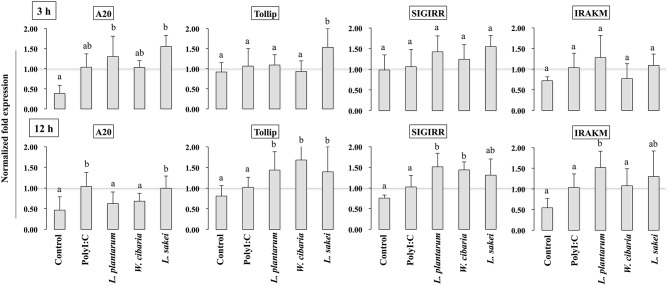
Analysis of TLRs negative regulators expression in HCT116 cells. After HCT116 cells treated LAB strains and PolyI:C for 3 and 12 h. The expression of A20, Tollip, SIGIRR, and IRAK-M were evaluated by RT-PCR. Cells treated with either PolyI:C or medium alone were used as positive and negative controls, respectively. The positive control was used for comparison of LAB strains treated groups The mean differences among the different superscript letters (a, b, ab) were significant at 0.05 level. LABs were able to modulate expression of negative regulators of TLR signaling in HCT116 cells, stimulated with PolyI:C.

To gain more insight into the mechanisms of LABs on modulation of innate antiviral immune responses of TLR signaling, we examined the expression of genes that negatively regulate TLR signaling in HCT116 that were treated with LABs and PolyI:C. The expression of A20, Tollip, SIGIRR, and IRAKM was analyzed by qRT-PCR. Stimulation of cells with PolyI:C increased the mRNA levels of A20, while it didn't up-regulate the levels of Tollip, SIGIRR, and IRAKM (Figure 8). The PolyI:C mediated expressions levels were able to modulate by LAB strains in a time dependent manner. At 3 h, *L. plantarum* and *L. sakei* treated HCT116 cells showed increase in level of A20, whereas the level of Tollip was only increased by *L. sakei*, and this increase was significantly higher (*p* < 0.05) than that induced by PolyI:C and other strains. But, all LABs weren't significantly increased the level of SIGIRR as compared to PolyI:C. In addition, there was no significant alternation observed in the level of IRAKM. In contrary, *L. plantarum* and *W. cibaria* reduced the level of A20 on 12 h, whereas all LABs showed significantly (*p* < 0.05) increase in levels of Tollip and SIGIRR as compared to PolyI:C (Figure 8). Furthermore, IRAKM mRNA expression was significantly increased by *L. plantarum* and *L. sakei*

### LABs Modulated the Phosphorylations of IRF3 and IκB-α in HCT116 Cells

**Figure 9:**
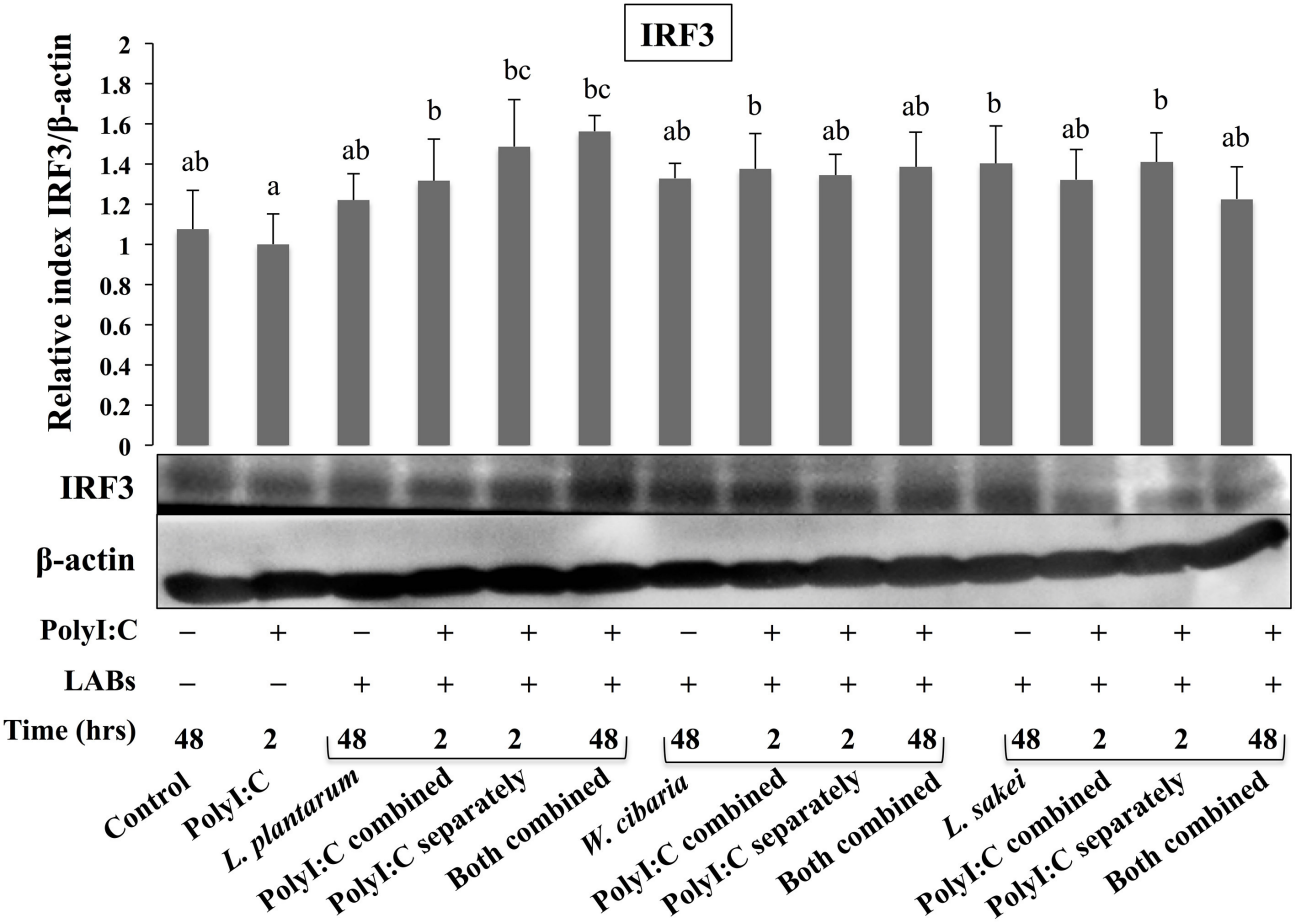
The ability of LABs to increase PolyI;C induced regulatory factor *in vitro*. HCT116 cells were stimulated as follows: control, PolyI:C alone, LABs alone (48 h), PolyI:C alone (2 h), LABs+PolyI:C (2 h, Poly:C combined treatment), LABs+PolyI:C (2 h, PolyI:C post-treatment separately), and or LABs+PolyI:C (48 h both combined). The phosphorylation of interferon regulatory factor 3 (IRF3) was analyzed by western blot. The loading control beta-actin was reused for illustrative purposes. The bar graphs are representative of three independent experiments. The mean differences among the different superscript letters (a, b, ab, bc) were significant at 0.05 level. LABs alone or co-treated with PolyI:C increased the phosphorylation of IRF3 in HCT116 cells.

**Figure 10:**
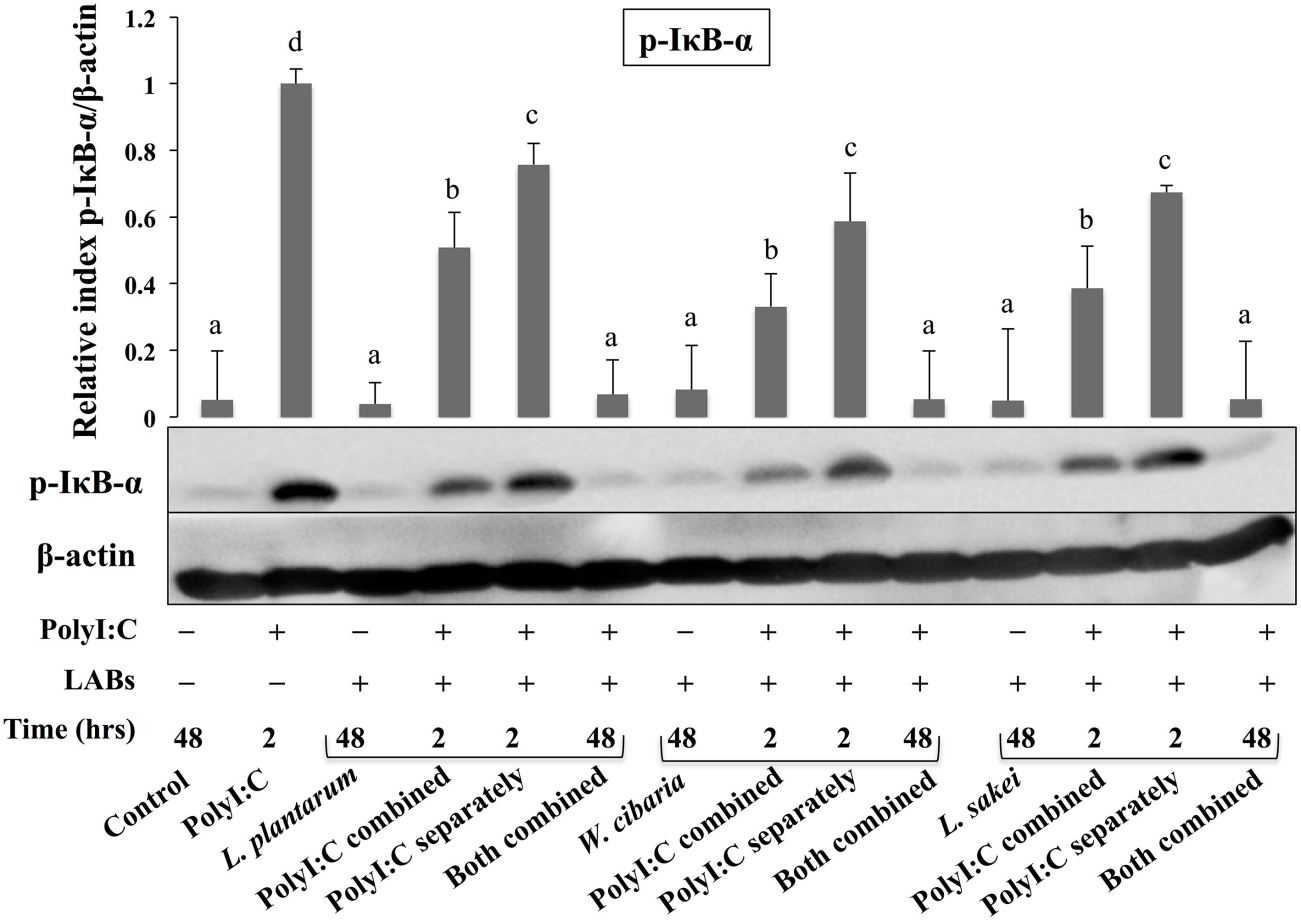
LABs inhibited PolyI;C induced activation of the NF-κB pathway *in vitro*. HCT116 cells were stimulated as follows: control, PolyI:C alone, LABs alone (48 h), PolyI:C alone (2 h), LABs+PolyI:C (2 h, Poly:C combined treatment), LABs+PolyI:C (2 h, PolyI:C post-treatment separately), and or LABs+PolyI:C (48 h both combined). The phosphorylation of IκB-α was analyzed by western blot. The loading control beta-actin was reused for illustrative purposes. The bar graphs are representative of three independent experiments. The mean differences among the different superscript letters (a, b, c, d) were significant at 0.05 level. LABs alone or co-treated with PolyI:C decreased the phosphorylation of IκB-α in HCT116 cells.

Activation of TLR3 by PolyI:C recruits several intracellular signaling molecules (TRAF3, IRF3, and NF-κB) to induce expressions of type 1 IFNs and inflammatory cytokines. Therefore, we examined whether LABs were able to modulate phosphorylation of IRF3 and IκB-α in HCT116 cells stimulated with PolyI:C. The level of p-IRF3 was increased when cells were treated with LABs in the presence of PolyI:C for 2 or 48 h (Figure 9). As compared to PolyI:C, LABs were able to increase the level of p-IRF3 in HCT116 cells post-treated with PolyI:C for 2 h. In contrary, PolyI:C alone significantly increased the phosphorylation of IκB-α in HCT116 cells, but cells treated with LABs alone or co-treated with LABs and PolyI:C for 48 h significantly decreased the level of IκB-α (Figure 10). In addition, stimulation of cells with LABs in the presence of PolyI:C for 2 h or post-stimulation with PolyI:C for 2 h diminished phosphorylation of IκB-α in HCT 116 cells as compared with PolyI:C alone. These results suggest LABs exhibits innate antiviral immune response by modulating the IRF3 and NF-κB pathways.

## Discussion

Intestinal epithelium contains IECs, which play important roles in maintenance of the intestinal immune system. Upon meeting pathogens, IECs capable to induce mucosal immune responses by expressing soluble factors such as cytokines/chemokines to recruit and activate immune cells including leukocytes and neutrophil granulocytes to the infected area, and by producing type I IFNs, antiviral proteins, and effector molecules to limit pathogens replications (25, 26). The expressions of TLRs in IECs play an important role on induction of mucosal immune responses in the intestine by sensing antigens derived from pathogens during their infection. Several studies have reported TLRs expressions and their vital roles on induction of host defense system against infectious diseases (4, 27). Activation of TLR3 by dsRNA induces production of several antiviral proteins such as IFN regulatory factors, type I IFNs, and cytokines/chemokines to establish antiviral state against viruses (28–30). Among the IFNs, IFN-β is a key cytokine that positively contributes to host innate immunity and defense against several viruses including rotavirus (31). In addition, the up-regulation of IFN-β induces transcription of other viral response genes involved in viral protection. IFN-β deficient mice have been shown to be more susceptible to influenza virus infection (32). Broquet et al. (33) reported that IFN-β showed protection against RVs by reducing their replication in IECs. Therefore, IFN-β is considered a potent antiviral chemokine, and thus, is used to screen or study the antiviral effects of immunobiotic strains and their related compounds. In this regard, we have evaluated whether LAB strains induce the expression of IFN-β in HCT116 cells. We found that LABs stimulation increased the expression of IFN-β in response to PolyI:C. Our findings are in good agreement with the results of others studies obtained for *Bifidobacterium infantis* MCC12 and *B. breve* MCC1274 (3), *Lactobacillus casei* MEP221106 (16), *Lactobacillus delbrueckii* TUA4408L (29), and *L. casei* Zhang (34). Probiotic *L. acidophilus* NCFM was able to increase the expression of IFN-β in bone marrow derived dendritic cells *in vitro* (35). Treatment of porcine IECs with *L. rhamnosus* CRL1505 induced expression of higher level of IFN-β, in response to PolyI:C (17). Moreover, probiotic *B. longum* SPM1206 and *L. ruminis* SPM0211 induced IFN-β expression in mice and Caco2 cells infected with RV (36).

In addition to the production of anti-viral cytokines, immunobiotics have been shown to improve protection against inflammatory condition or viral infection by regulating the expression of pro-inflammatory cytokines/chemokines (3, 16). We also found that LABs were able to reduce expression of pro-inflammatory cytokines (IL-6, IL-8, MCP-1, and IL-1β) in HCCT116 cells stimulated with PolyI:C. Production of these cytokines has also been reported to play crucial roles in host innate response against virus infections. The expression of pro-inflammatory cytokines (IL-6 and IL-8) was up-regulated in epithelial cells infected with RVs (37, 38), and infection of HT-29 cells with RVs increased the IL-8 level, which is dependent on protein kinase activity and NF-κB activation (37, 38). Clemente et al. (39) found that the infection of epithelial cells with RVs induced production of IL-6 and IL-8 via activation of the MAPK pathway. Porcine IECs cells treated with *L. acidophilus* and *L. rhamnosus* GG reduced the level of IL-6 and mucin, and increased TLR2 level in response to RV (22).

During the host infected with virus, the activation of TLR signaling increases expression of type I IFNs, which induces transcription of several IFN-stimulated genes (ISGs) that encode proteins with potent antiviral effector functions to block viral replication (40). MxA and RNase-L proteins are promising IFN-induced proteins with broad anti-viral activity against several different DNA and RNA viruses (13, 41). Myxovirus resistance protein (MxA) mainly targets viral nucleoproteins for its attachment, and reduces replication and intracellular proliferation of viruses. RNase L (2′-5′ oligoadenylate dependent endoribonuclease) is believed to contribute role in the anti-viral activity of IFNs and the stability of IFN-induced genes such as ISG (13). 2-5A oligoadenylate synthetase (OSA), especially OSA1, activates the latent form of RNase L, and thus, causes the cleavage of viral RNA and inhibits viral replication and proliferation. Furthermore, viral protein (VP3) derived from RVs has been shown to inhibit RNase L activity by cleaving 2-5A (42). Previous studies reported that immunobiotics have the ability to improve expression of these IFN stimulated antiviral proteins (MxA and OAS) along with higher level of IFN-β (3, 43). We also found that cells stimulated with LABs increased MxA and OAS1 levels in response to PolyI:C. Similarly, *L. delbrueckii* TUA4408L and *B. infantis* MCC12 increased the expression of these antiviral proteins in bovine and porcine IECs (29, 43). Viruses have evolved mechanisms to eradicate host immune system by interacting with PRR receptors that mediate signaling cascades to develop antiviral immunity (41). RIG-1 and TLR3 are RRR receptors that can initiate signaling cascades for IFN-β up-regulation (44) The pre-exposure of cells to LABs increased the expressions of RIG-1 and TLR3, which confirmed the antiviral effects of LAB strains *in vitro* (3).

The intestinal barrier is a tight structure that provides protection against harmful environments, but it has been reported to be dysfunctional that associated with paracellular permeability in several diseases. Tight-Junction (TJ) proteins are the responsible components that connect IECs with neighbor cells and control paracellular gut permeability (45). Administration of PolyI:C has been shown to induce severe mucosal damage in the mouse small intestine of mice, which is probably due to the activation of TLR3 signaling by PolyI:C (46). Our study showed that the presence of LAB strains increased the level of TJ proteins (ZO-1 and occluding), indicating that LABs have the ability to maintain gut-barrier integrity and reduce gut dysfunction. Intestinal epithelium acts as a mucosal barrier that mediates signaling to underlying immune cells by sensing antigens and intestinal changes (47). Cross-talk between these two cells is an important feature that helps maintain the mucosal barrier and provides protection against infectious diseases and harmful environments. In a Transwell experiment, basolateral treatment of RAW264.7 cells with lipopolysaccharide increased the productions of TNF-α and IL-8 in Caco2 cells that were taken in apical side (48). Another *in vitro* study, it was reported that in response to commensals, IECs were able to stimulate underlying dendritic cells (DCs) by secreting several inflammatory proteins (49). TNF-α is a pleiotropic pro-inflammatory cytokine, which has been shown to play critical roles in the pathogeneses of several diseases, including viral infections. In response to PolyI:C, bone marrow derived macrophages, Raw264.7, and THP-1 cells increased TNF-α production *in vitro* (50, 51). IL-10 is a prime anti-inflammatory cytokine that plays key roles in the maintenance of gut hemostasis and innate immunity and in the pathogenesis of IBD (52). Through co-culture study, we found that LAB strains improved the cross-talk between IECs and THP-1 cells, which resulted in decreased levels of pro-inflammatory cytokines (TNF-α, IL-1β) and increased levels of anti-inflammatory cytokine (IL-10) and INFs. These results were consistent with those of other study (53), in which HT-29 cells stimulated with three probiotics strains (*L. helveticus* R0052, *B. longum* subsp. *infantis* R0033, *B. bifidum* R0071) decreased the level of TNF-α and IL-8 in response to PolyI:C.

**Figure 11:**
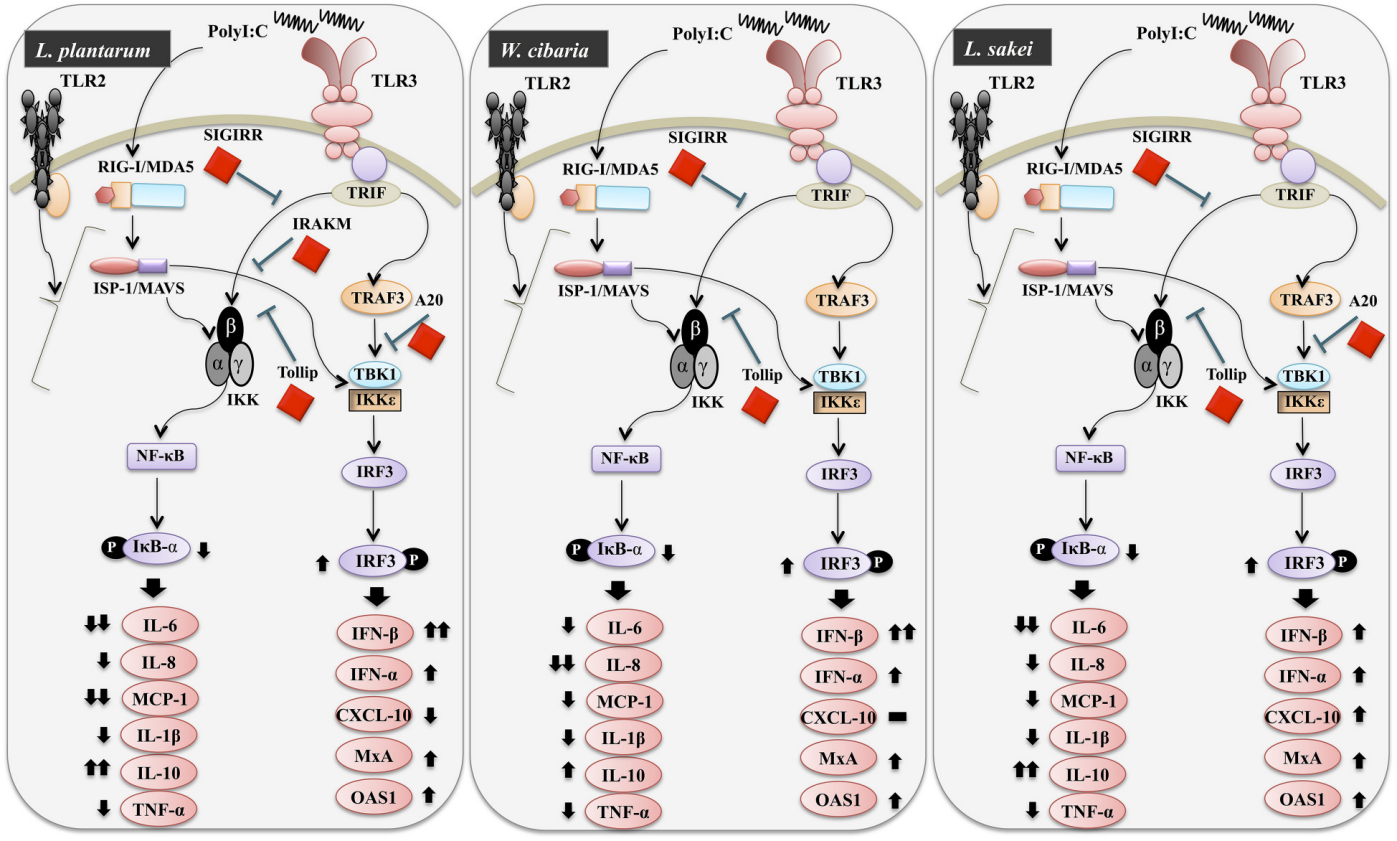
Schematic model for the mechanisms involved in the antiviral activity of *L. plantarum, W. cibaria*, and *L. sakei in vitro*.

TLR activation is an important process that could be involved in the development of infectious diseases. Once it activated, several intracellular proteins actively participate in control of hyper-activation of TLR signaling pathways by negatively regulating the transcription of TLR genes. Tollip, SIGIRR, A20, and IRAK-M are the potent negative regulators that have been shown to attenuate over activation of TLR signaling in IECs (54). High levels of Tollip may prevent inflammatory cytokine production by commensal bacteria in the gut (55), and the knockdown of Tollip increased the expression of inflammatory cytokines and activation of NF-κB in Caco-2 cells (56). SIGIRR is a transmembrane receptor that may be expressed by IECs and immature DCs derived from gut. Polentarutti et al. (57) reported that SIGIRR overexpression could inhibit IL-1 and IL-18 mediated NF-κB activation in DCs, and thus, it playing a role in the regulation of intestinal inflammatory response. In another study, the expression of SIGIRR in IECs attenuated exaggerated inflammatory response and promoted commensal bacteria colonization against intestinal pathogens, indicating a close relationship exists between commensal bacteria, and IECs (4). A20 is a zinc-finger protein and its knockdown in mouse macrophage increased the inflammatory cytokine expressions in response to TLR2 and TLR3 ligands (4). In addition, the presence of A20 has been reported to suppress TLR3-mediated activation of IRF3 in transfected cells (58). In view of these observations, we evaluated the expressions of TLR negative regulators in HCT116 cells stimulated with PolyI:C. Exposure of cells to LABs were able to modulate expression of negative regulators of TLR signaling in HCT116 cells, which indicate LAB mediated attenuation of inflammatory response *in vitro*. Similar results were observed in other studies for *B. infantis* MCC12 and *B. breve* MCC1274 (3), *L. delbrueckii* OLL1073R-1 (18), and *L. delbrueckii* TUA4408L (29). During viral infections, PRRs of host cells activate IFN regulatory factors (IRFs) that regulate the production of IFN-β (44). In particular, NSP-1 (a non-structural protein of RVs) has been reported to have high affinity for IRF3, which results in proteasome-dependent degradation of transcription factors (59). In the present study, as we expected, challenging of HCT116 cells with LABs increased the phosphorylation of IRF3 in response to TLR3 agonist. Furthermore, NF-κB pathway is an important TLR signaling pathway and its activation increases the production of inflammatory cytokines *in vitro* and *in vivo* (60). Therefore, we also analyzed activation of the NF-κB pathway in HCT116 cells. We found that LAB strains down-regulated p-IκB-α, indicating that LABs were able to attenuate PolyI:C induced viral and inflammatory response by modulating IRF3 and NF-κB pathways (Figure 11). Similar results were obtained by Kim et al. (30), who reported that lipoteichoic acid of *L. plantarum* attenuated PolyI:C mediated NF-κB activation in porcine IPEC-J2 cells.

In conclusion, our study demonstrated that *in vitro* exposure of different LAB strains beneficially modulates innate antiviral immune responses induced by PolyI:C in HCT116 cells. LABs significantly up-regulated the mRNA level of IFN-β and down-regulated IL-6, IL-8, MCP-1, and IL-1β levels by modulating the expressions of negative regulators of TLRs and the activations of IFR3 and NF-κB pathways. Our results also show that cells co-treated with LABs and PolyI:C improved the gut barrier integrity by maintaining of TJ proteins *in vitro*, suggesting LABs might protect IECs from harmful environments *in vivo*. Furthermore, our co-culture study showed that LABs indirectly modulated TLR3 triggered innate antiviral response in monocyte-derived macrophages. Overall, LABs protected cells from PolyI:C *in vitro*, and an additional study will be performed to determine whether these findings are duplicated *in vivo*.

## Data Availability

All datasets generated for this study are included in the manuscript and/or the Supplementary Files.

## Author Contributions

PK and HK designed the study and assisted with the experiments and interpretation of results. PK performed the assays and wrote the manuscript. All authors read and approved the final manuscript.

### Conflict of Interest Statement

The authors declare that the research was conducted in the absence of any commercial or financial relationships that could be construed as a potential conflict of interest.
