# Supplementary material for: Immunobiotic Strains Modulate Toll-Like Receptor 3 Agonist Induced Innate Antiviral Immune Response in Human Intestinal Epithelial Cells by Modulating IFN Regulatory Factor 3 and NF-κB Signaling
Source: Front Immunol. 2019 Jul 3;10:1536. doi: 10.3389/fimmu.2019.01536 (PMC6618302; doi:10.3389/fimmu.2019.01536)
Supplement: Supplementary file 1 [file Presentation_1.pptx]

## Slide 1
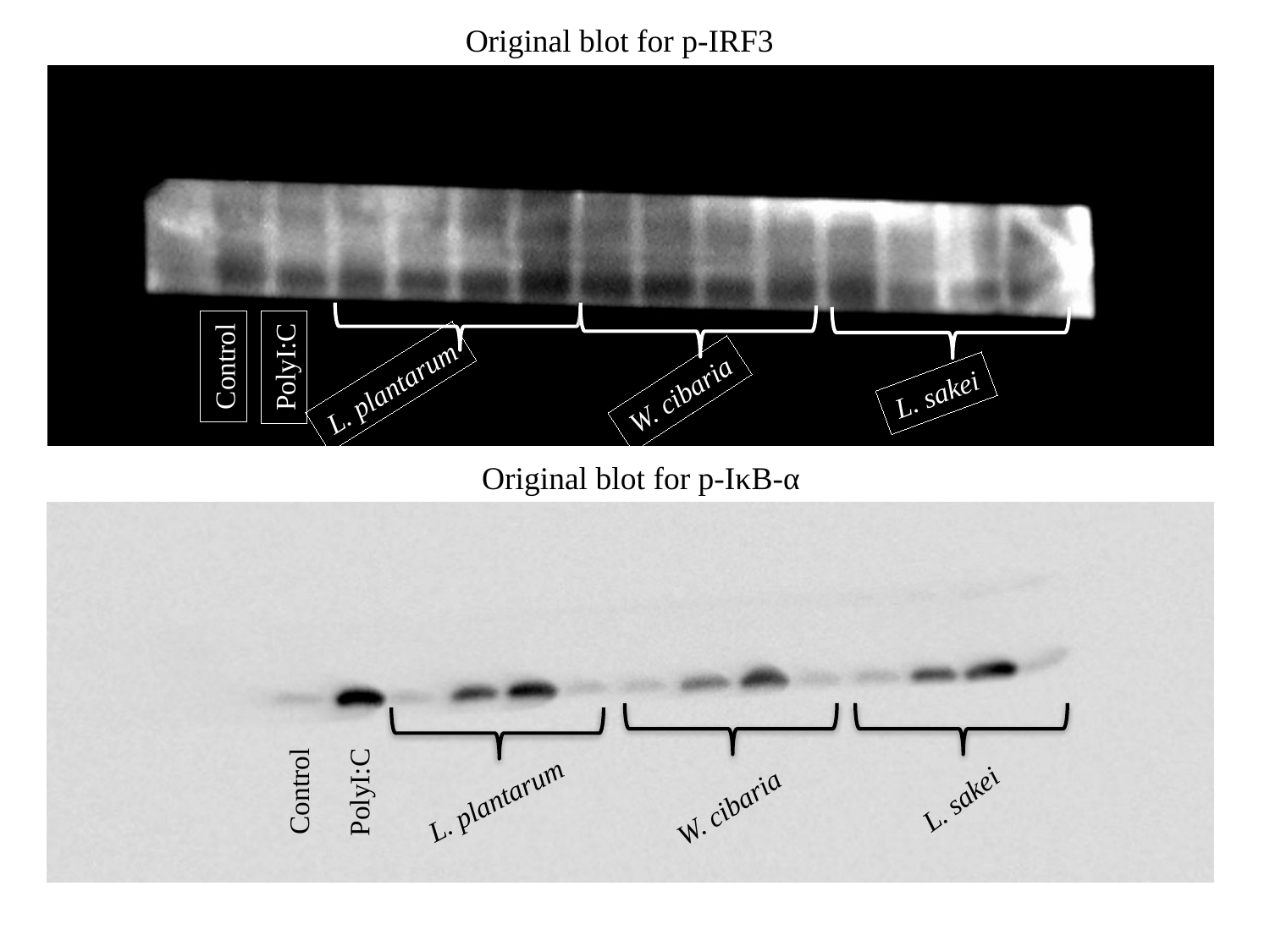

Original blot for p-IRF3
Control
PolyI:C
L. plantarum
L. sakei
W. cibaria
Original blot for p-IκB-α
Control
PolyI:C
L. sakei
L. plantarum
W. cibaria

## Slide 2
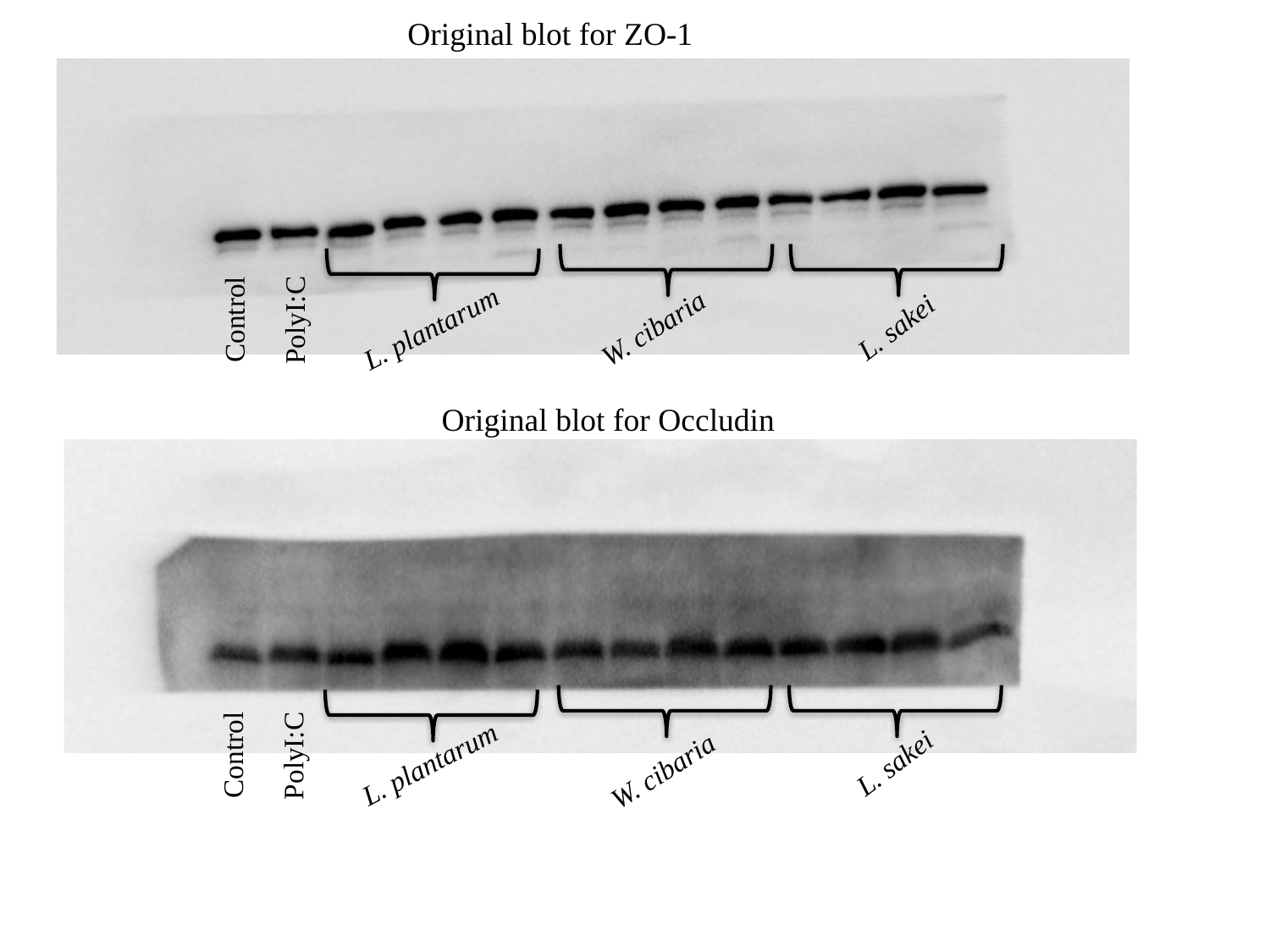

Original blot for ZO-1
Control
PolyI:C
L. sakei
L. plantarum
W. cibaria
Original blot for Occludin
Control
PolyI:C
L. sakei
L. plantarum
W. cibaria

## Slide 3
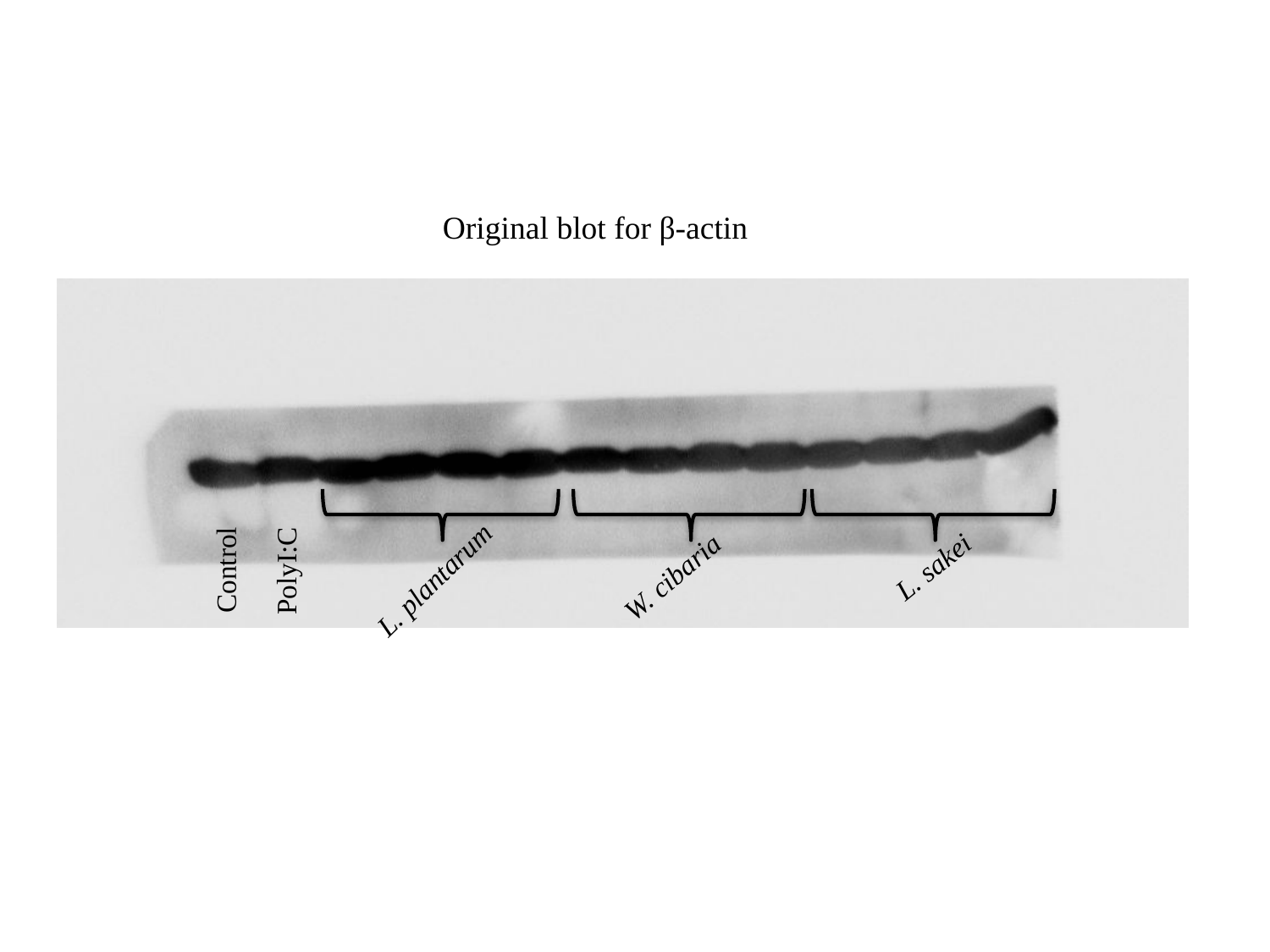

Original blot for β-actin
L. sakei
Control
PolyI:C
W. cibaria
L. plantarum
